# Follow up of the Humoral Response in Healthcare Workers after the Administration of Two Dose of the Anti SARS-CoV-2 Vaccines—Effectiveness in Delta Variant Breakthrough Infections

**DOI:** 10.3390/v14071385

**Published:** 2022-06-24

**Authors:** Gema Fernández-Rivas, Jaume Barallat, Bibiana Quirant-Sánchez, Victoria González, María Doladé, Eva Martinez-Caceres, Monica Piña, Joan Matllo, Ignacio Blanco, Pere-Joan Cardona

**Affiliations:** 1 Microbiology Department, Clinical Laboratory North Metropolitan Area, Germans Trias i Pujol University Hospital, 08916 Badalona, Spain; vgsoler@iconcologia.net (V.G.); pcardonai.germanstrias@gencat.cat (P.-J.C.); 2 Department of Genetics and Microbiology, Autonomous University of Barcelona, 08916 Badalona, Spain; 3 Biochemistry Department, Germans Trias i Pujol University Hospital, 08916 Badalona, Spain; jbarallat.germanstrias@gencat.cat (J.B.); mdolade.germanstrias@gencat.cat (M.D.); 4 Immunology Department, Clinical Laboratory North Metropolitan Area, Germans Trias i Pujol University Hospital, 08916 Badalona, Spain; bquirant@gmail.com (B.Q.-S.); emmartinez.germanstrias@gencat.cat (E.M.-C.); 5 Center for Epidemiological Studies on Human Immunodeficiency Virus Infection and Acquired Immunodeficiency Syndrome (HIV/AIDS) and Sexually Transmitted Infections (STI) of Catalonia (CEEISCAT), Generalitat de Catalunya, 08916 Badalona, Spain; 6 Centro de Investigación Biomédica en Red (CIBER) in Epidemiology and Public Health (CIBERESP), 28029 Madrid, Spain; 7 Department of Prevention and Risks, Primary Care Management, Northern Metropolitan Territorial Management, Catalan Health Institute, 08916 Badalona, Spain; mpinar.mn.ics@gencat.cat; 8 Department of Prevention and Risks, Germans Trias i Pujol University Hospital, Northern Metropolitan Territorial Management, Catalan Health Institute, 08916 Badalona, Spain; jmatlloaguilar@gencat.cat; 9 Metropolitana Nord Laboratory, Germans Trias i Pujol University Hospital, 08916 Badalona, Spain; iblanco.germanstrias@gencat.cat

**Keywords:** humoral response, SARS-CoV-2, Delta variant

## Abstract

The implementation of vaccination among healthcare workers (HCWs) allowed the management of the pandemic in a manner that differed from that in the first waves. It has been demonstrated that the mRNA vaccines elicit good humoral responses but that there are still breakthrough infections. In summer 2021, a fifth wave emerged, despite the good coverage of HCWs in Spain. We aimed to study the SARS-CoV-2 IgG antibody levels as a marker to predict the possibility of Delta variant infections after vaccination after a seroepidemiological campaign. Of the 5000 participants, a total of 4902 (98.04%) showed a positive result in the serological anti-S test and only 98 (1.96%) were negative. Among the 4368 fully vaccinated participants, only in five cases was the serology negative. Of the total number of participants that received antibody results during the study, 162 were PCR positive in the subsequent two months. Among these, 151 were fully vaccinated (two doses). Significant differences between antibody BAU/mL levels were found between PCR positive and non-PCR positive participants (*p* < 0.01). The median of BAU/mL was higher in those vaccinated patients with no infection (1260 BAU/mL; 465–2080) versus infected patients (661 BAU/mL; 361–2080). These data support the idea that vaccines play an important role in the control of the pandemic, especially among HCWs at the time of the Delta variant circulation. More studies with other variants of concern must be performed in order to establish a correlation between the levels of IgG and the new infections.

## 1. Introduction

The introduction of vaccination in HCWs has been a turning point in the epidemiology of COVID-19. Complete vaccination protects, with high efficiency, against severe COVID-19, but displays variable protection against infection, depending on diverse factors, such as time post-vaccination or the SARS-CoV-2 predominant variant. Current data from the Comirnaty vaccine (Pfizer/BioNTech) show a significant increase in anti-S IgG at 21 days post-first dose and a maximum and a sustained peak at the seventh day after the second dose. These vaccines induce the production of specific neutralizing antibodies associated with protective immunity. The humoral response to mRNA vaccines based on neutralizing antibody assays has been described in other published trials and articles [1,2].

Few studies describe the durability of anti-S antibodies after the administration of the mRNA vaccines, developed by Pfizer/BioNTech and Moderna, showing that the neutralizing antibody titers persist at least 6 months after the second dose with different serological tests [2,3]. It has also been possible to study the response of other types of vaccines such as those using Adenovirus vectors (Ad26.COV2.S vaccine (Johnson & Johnson-Janssen)). In this case, the vaccine-elicited long-lasting humoral and cellular immune responses with minimal decreases for at least 8 months after immunization [4]. In addition, an increase in the neutralizing antibody titer against different variants of SARS-CoV-2, including against variant B. 1.617.2, which is more transmissible, and variants B.1.351 and P.1, which are partially resistant to neutralization, suggests maturation of B cell responses even without any additional boost [5]. Even Heterologous SARS-CoV-2 vaccination showed high titers of SARS-CoV-2 antibodies and is comparable with homologous vaccination regimens [6].

Achieving a high rate of vaccination coverage during 2022 seems plausible in many countries. After vaccination, antibodies can be detected by conventional serological techniques in most individuals, with few exceptions. This is the case of some immunocompromised patients, including those with primary or secondary immunodeficiencies, e.g., patients treated with immunosuppressive drugs [7,8,9].

Especially important is the study of HCWs since it is essential to know their level of protection whose exposure to the virus is higher than the general population [2,10,11]. In this context, after transversal studies, a percentage of HCWs do not have positive titers of IgG anti-S antibodies using screening standard techniques [5,12]. In those cases, a broader study of the immune response, with the analysis of specific T-cell proliferation and/or cytokine production in front of SARS-CoV-2 peptides, is needed. SARS-CoV-2 specific T cells can be detected using peptide pools in various T cell assays in both COVID-19 infected and vaccinated patients [13,14].

There is a need to understand the roles of SARS-CoV-2 T cell responses as a potential factor associated with disease outcome and/or vaccine protection against severe disease [15]. Recent evidence suggest that SARS-CoV-2 infection generates a largely novel repertoire of T cells [16,17]. This immunological information can be used to minimize the risk of exposure and spread.

In May 2020, a seroprevalence study in HCW of the Northern Metropolitan Area of Barcelona was performed to estimate the incidence of SARS-CoV-2 infections, with 10.3% of results positive for anti-SARS-CoV-2 IgG (specific for either S1/S2 or N antigens) [18].

Since the beginning of the COVID-19 pandemic, it had been assumed that SARS-CoV-2 infection would protect from reinfection and that neutralizing antibodies would correlate with protection, but progress in pandemic control is slowed by the appearance of variants that seem to be more transmissible and capable of avoiding neutralizing antibodies. In addition, neutralizing antibody quantification is a time-consuming technique that is not available in many hospitals and is difficult to scale up. Recently, automated chemiluminescence methods for SARS-CoV-2 trimeric IgG antibodies have been standardized using WHO reference standards [19]. We hypothesize that the measurement of total SARS-CoV-2 IgG antibodies may be a marker to predict the possibility of infections after vaccination. These breakthrough infections have been reported in many countries. Most of them were mild or asymptomatic and, therefore, they may contribute to the spread of the disease among HCWs and patients.

Thus, the aim of this study was to assess the coverage of the COVID-19 vaccines and to estimate the protection in the fifth wave regarding new infections with the new variants of concern (VOC) in HCWs in the Northern Metropolitan Area of Barcelona.

## 2. Material and Methods

From 1 June 2021, all HCWs of the ICS-Northern Metropolitan Area of Barcelona (*n* = 9315) were offered to have serum testing performed for SARS-CoV-2 trimeric IgG antibodies as a service to determine the post-vaccine serological status. Participation in this study was voluntary; HCWs were not selected. All individuals willing to participate fulfilled a brief epidemiological questionnaire that included demographic data, professional information, and a direct question about whether they had been diagnosed with COVID-19, type of vaccine, date of vaccination, and if they had received one or two doses.

**Figure 1:**
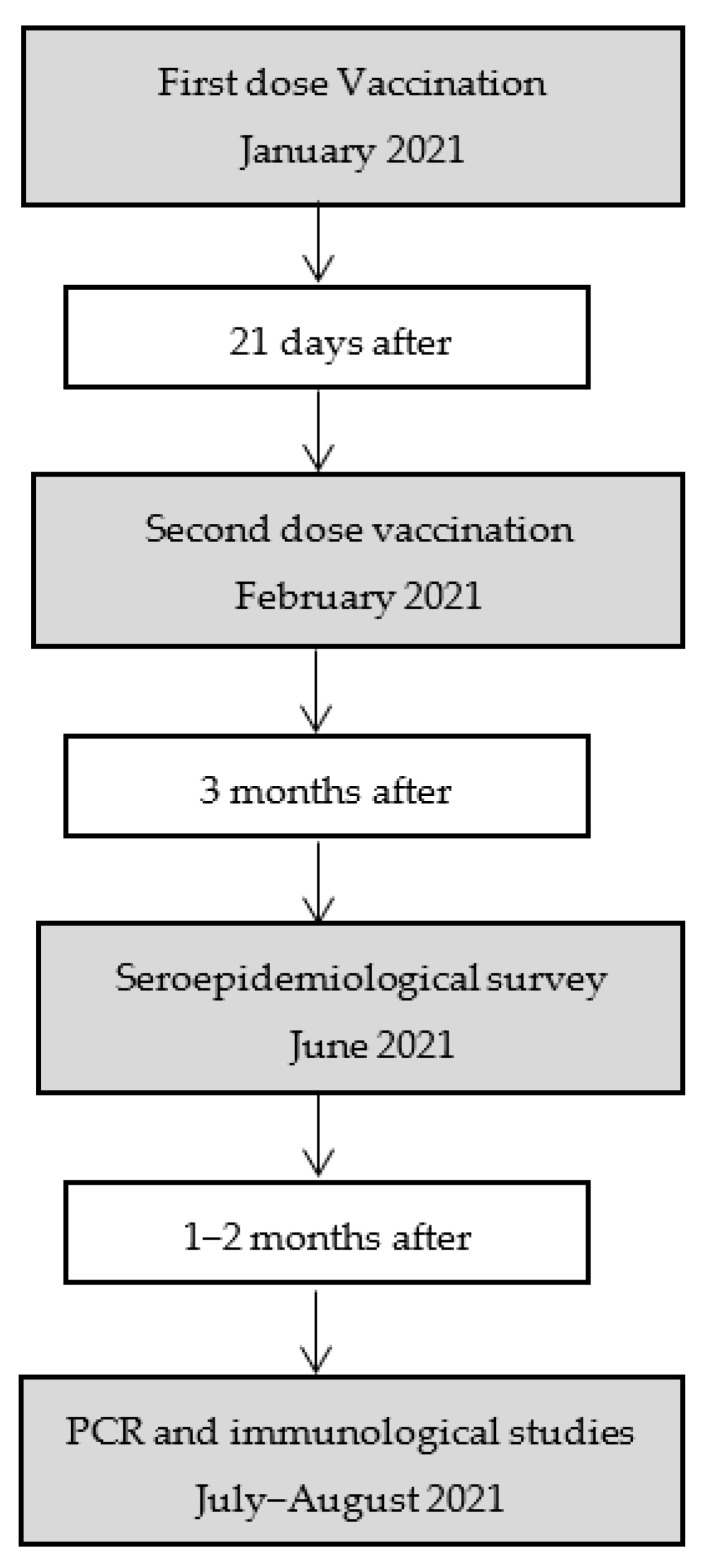
Flowchart of the process of the study.

From 1 January 2021 to the time of writing this paper, all HCWs were offered vaccination with mRNA vaccines, with an actual coverage of 94.11% (*n* = 4518) with at least 2 doses and 96.96% (*n* = 4655) with at least one dose. From 1 June 2021, a follow-up transversal study of seroprevalence was offered to all of them, regardless of their vaccination status, in a first approach to assessing the immunological response to the vaccines. Due to the occurrence of the fifth pandemic wave in June–July 2021 in the North Barcelona Metropolitan region, we studied the relationship between the antibody BAU/mL level and the COVID-19 infection. Policies for PCR determination in vaccinated individuals included close contacts and symptomatic patients. The circulation of the Delta variant (B.1.617.2 linage) was predominant in that period. The process of the study is summarized in Figure 1.

The study obtained ethical approval from the Ethics Committee of Germans Trias i Pujol Hospital (PI-21-291). All participants gave written informed consent in case additional immunological studies were required (fully vaccinated participants with negative Anti-S antibodies).

In case of positive PCR after serology study, symptoms were also recorded. Symptoms were classified into 6 categories: 1—‘flu-like’ with no fever: headache, loss of smell, muscle pains, cough, sore throat, chest pain, no fever; 2—‘flu-like’ with fever: headache, loss of smell, cough, sore throat, hoarseness, fever, loss of appetite; 3—gastrointestinal: headache, loss of smell, loss of appetite, diarrhea, sore throat, chest pain, no cough; 4—severe level one, fatigue: headache, loss of smell, cough, fever, hoarseness, chest pain, fatigue; 5—severe level two, confusion: headache, loss of smell, loss of appetite, cough, fever, hoarseness, sore throat, chest pain, fatigue, confusion, muscle pain; 6—severe level three, abdominal and respiratory: headache, loss of smell, loss of appetite, cough, fever, hoarseness, sore throat, chest pain, fatigue, confusion, muscle pain, shortness of breath, diarrhea, abdominal pain [20].

### 2.1. Laboratory Analysis

Serum testing was conducted by the Regional Clinical laboratory using the quantitative SARS-CoV-2 Trimeric IgG LIAISON XL test (DiaSorin, Vercelli, Italy) on the LIAISON XL platform, following the manufacturer’s instructions. Antibody concentrations are expressed as Binding Antibody Units (BAU)/mL, which are referenced concerning the First International Standard of the WHO for immunoglobulin against SARS-CoV-2 (20/136) [17]. The LIAISON^®^ SARS-CoV-2 TrimericS IgG discriminates among negative (<33.8 BAU/mL) and positive (≥33.8 BAU/mL) samples.

SARS-CoV-2 infection was defined as testing positive for SARS-CoV-2 via a PCR or TMA test from any sample (i.e., bronchial lavage, nasopharyngeal or nasal swab, oropharyngeal swab, throat swab, saliva, sputum, or tracheal aspirate) in any clinical setting. Upper respiratory swabs for diagnosis of SARS-CoV-2 infection in HCW with clinical symptoms of COVID-19 were processed by transcription-mediated amplification (TMA, Procleix^®^ SARS-CoV-2 assay, Griffols, Barcelona, Spain) according to the manufacturer’s instructions or by RT-PCR (Allplex 2019-nCoV Assay, Seegene, Korea).

Participants who had negative anti-spike IgG antibodies were further studied, and the immunoglobulin levels and peripheral lymphocyte subpopulations were analyzed by flow Cytometry and SARS-CoV-2 proliferative T cell responses.

IgA, IgG, and IgM were analyzed on an AU5800 Chemistry Analyzer (Beckman Coulter, Inc., Brea, CA, USA) with reagents from the same manufacturer. The tests were performed according to the manufacturers’ recommendation.

To analyze lymphocyte subpopulations, whole blood (100 μL) was incubated with a mix of specific conjugated monoclonal antibodies (mAb) (CD3 PerCP, CD4/CD8 FITC/PE, CD45 APC, CD19 V500, CD56 V450, CD16 V450, BD Bioscience, San Jose, CA, USA) and gently mixed for 20 min at room temperature (RT) in the dark. Samples were treated with 1 mL of lysing solution (BD FACS lysing solution, Beckton-Dickinson, San Jose, CA, USA), vortexed, and incubated for 15 min at RT in the dark. Samples were then washed with phosphate-buffered saline (PBS), stored at RT in the dark, and analyzed within 1 h; the samples were acquired with a flow cytometer. At least 100,000 events were acquired from each sample.

SARS-CoV-2 T cell reactivity was performed with a dye-based proliferation assay [18]. Briefly: Blood samples were obtained in 10 mL sodium heparin tubes (BD Vacutainer, Beckton-Dickinson, San Jose, CA, USA)) and peripheral blood mononuclear cells (PBMC) were separated by ficoll density gradient centrifugation within 4 h after the extraction. PBMC were resuspended in a phosphate-buffered saline (PBS) solution up to a concentration of 8 × 10^6^ cells/mL, stained with 1µL Violet Proliferation Dye 450 (VPD-450) (BD Bioscience) per ml of suspension and incubated at 37 °C, in the dark for 12 min. The cell culture medium was prepared with RPMI medium (GIBCO, Thermo Fisher Scientific, Waltham, MA, United States) supplemented with glutamine, penicillin, streptomycin, and 5% of patient’s serum obtained with 5 mL serum tubes (BD Vacutainer). A mix of 15-mer peptides covering the complete sequence of nucleocapsid phosphoprotein (N), the complete sequence of membrane glycoprotein (M), and the sequence domain aa 689–895 from the spike (S) (PepTivator^®^ SARS-CoV-2, Miltenyi Biotec, Bergisch Gladbach, Germany) was prepared for the PBMC culture. Cells were finally cultured in 96 well plates at a concentration of 150,000 cells/well for 7 days at 37 °C. A minimum of 12 wells were cultured with the mix of peptides at a concentration of 0.6 nmol/mL each peptide, 12 wells of negative control without proliferation stimulus, 4 positive controls with Phytohaemagglutinin P (PHA), and 4 positive controls with cytomegalovirus peptides (PepTivator^®^ CMV pp65, Miltenyi Biotec). After culture, cells were labeled with 7-aminoactinomycin D (7-AAD) (BD Bioscience) to assess cell viability, and with anti-CD3-APC (Biolegend, San Diego, CA, USA), anti-CD4-PC (Biolegend, San Diego, CA, USA), and anti-CD8-FITC (Biolegend) for T cell analysis. Cell culture was considered positive when proliferation was superior to the median of negative controls proliferation plus three standard deviations within the CD3 population. SARS-CoV-2 T cell reactivity from individuals without SARS-CoV-2 infection or vaccination is absent.

Samples were acquired with FACS Lyrics (BD Bioscience,) and analyzed using FACS Diva software (BD Biosciences).

### 2.2. Statistical Analysis

Categorical variables are expressed as frequencies. Quantitative variables were classified by a Kolmogorov–Smirnoff normality test. Parametric variables are expressed as the mean and standard deviation and non-parametric variables as median and interquartile range [IQR]. Quantitative variables were compared using the Mann–Whitney U Test. The statistical analysis was performed by using the SPSS v20 statistical package (IBM Corp., Armonk, NY, USA).

## 3. Results

A total of 5000 healthcare workers from the Northern Metropolitan Area of Barcelona participated in the study. The participation rate was 53.68% (5000/9315). A total of 4902 (98.04%) showed a positive result in the serological anti-S test and only 98 (1.96%) were negative. Demographic and professional characteristics of the participants are shown in Table 1.

Of the participants with one dose vaccination (*n* = 166), 113 (67.64%) had a previous COVID-19 diagnosis, and 103 (61.67%) showed antibody detection superior to the quantification limit (>2080 BAU/mL). In those with a complete vaccination scheme (*n* = 4368), 1812 (41.19%) showed levels of antibodies > 2080 BAU/mL.

A total of 4902 participants of the serological study were SARS-CoV-2 Trimeric IgG-positive after the performance of the antibody test. Among the 4368 fully vaccinated participants, only in five cases was the serology was negative. Among these five vaccinated participants with negative serology, a minimum of 40 days had passed between the second dose and blood sample extraction.

**Figure 2:**
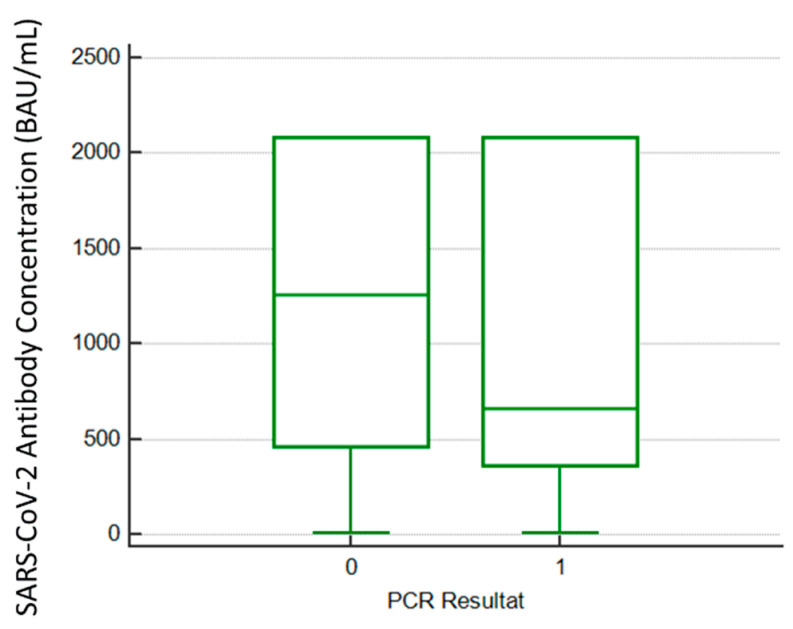
Differences in median BAU/mL SARS-CoV-2 previous antibody levels between 0 = Negative and 1 = Positive HCWs. In both cases the upper range corresponds to the limit of quantification of our assay (2080 BAU/mL).

Of the total participants that had antibody results during the month of June, 162 were PCR-positive in July or August. Among these, six were not vaccinated, five had received only one dose (4/5 were previously diagnosed with COVID-19) and 151 were fully vaccinated. None of them required hospitalization. Significant differences between antibody BAU/mL levels were found between PCR-positive and non-PCR-positive participants when the Mann–Whitney U test was performed (*p* < 0.01). The median of BAU/mL was higher in those vaccinated patients with no infection (1260 BAU/mL; 465–2080) versus the infected patients (661 BAU/mL; 361–2080) (Figure 2). No differences among ages nor sex were regarded (*p* > 0.05).

**Figure 3:**
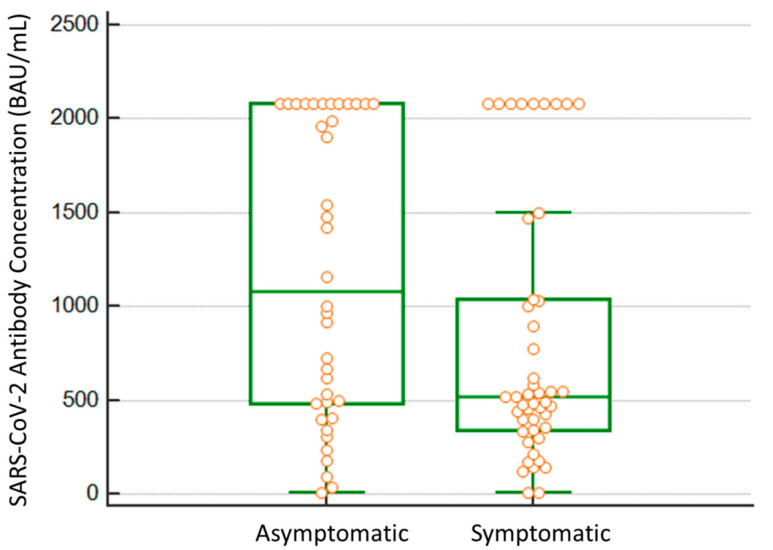
Differences in median BAU/mL SARS-CoV-2 previous antibody levels between infected HCWs that were recorded as not symptomatic (*n* = 40) and symptomatic (*n* = 45). All single dots are overlaid on the box and whisker plot, as the number of outliers is remarkable.

Additionally, in 85 PCR-positive hospital HCWs, symptoms were recorded and differences between symptomatic and asymptomatic individuals were analyzed. Significant differences were observed in the Mann–Whitney U test (*p* = 0.038). Symptomatic participants had lower antibody levels compared to asymptomatic HCW (515 BAU/mL; 339–1040 vs. 1080 BAU/mL, 465–2080) (Figure 3).

Regarding the type of symptoms, no severe COVID-19 patients were identified. Symptom classification is depicted in Table 2.

**Figure 4:**
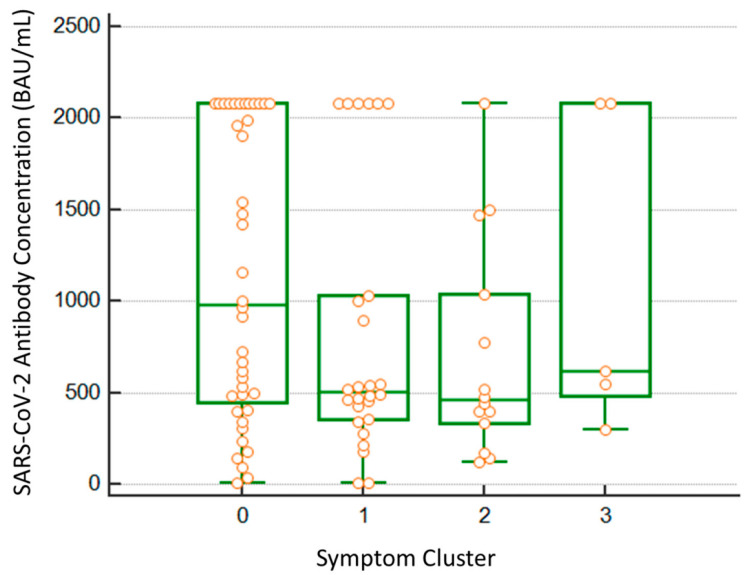
Differences in median BAU/mL SARS-CoV-2 previous antibody levels between four groups. 0: Non-symptomatic (*n* = 40); 1: ‘Flu Like’ with fever (*n* = 26); 2: ‘Flu-Like’ no fever (*n* = 14); 3: Gastrointestinal symptoms (*n* = 5). All single dots, including outliers, are overlaid on the box and whisker plot.

The number of participants in each category was not considered adequate for significant statistical analysis. Nevertheless, a tendency to have lower antibodies prior to infection in flu-like clusters was observed, as depicted in Figure 4.

In order to investigate the immune response after vaccination in those participants who showed negative anti-spike antibodies, we carried out an extended immunological study of lymphocyte subpopulations, immunoglobulin levels, and SARS-CoV-2 T cell reactivity. Only two seronegative participants were available for extended study. Both participants showed a reduced absolute number of both CD4 and CD8 T cells, and B lymphocyte subpopulations and decreased IgG levels. However, SARS-CoV-2 T cell reactivity was positive against spike protein (Table 3).

## 4. Discussion

Due to the knowledge of anti-S antibodies to SARS-CoV-2 and the concomitant arrival of the fifth wave, it has been possible to study the relationship between the level of antibodies and the development of breakthrough infections in vaccinated HCWs by the Delta variant. In this study, we found a relation between the possibility of developing the infection and the level of total IgG anti-S antibodies below 661 BAU/mL.

The role of HCWs has been crucial for pandemic control. Since January 2021, the public health authorities understood that vaccination of HCWs was a priority for a strong health system. A tremendous effort was undertaken to proceed with a total vaccination among this collective. Controversy about the need for testing antibody response after vaccination was discussed; in fact, health authorities did not recommend it [21]. Despite this fact, the study of anti-S IgG was well accepted among HCWs and high participation was observed. The seroprevalence study was conducted in order to assess the antibody titers 4 months after vaccination and to understand the effect of the fifth wave in the HCW in our settings.

The main concern regarding SARS-CoV-2 vaccination is the establishment of durable protection against infection and severity [22]. Based on the evidence from several waves following vaccination, it seems that antibody response is not as conserved as in other types of vaccines, and effectiveness wanes over time. This may be related to lower protection from reinfection, as antibodies play a main role in the neutralization of viruses. The majority of HCW (40.38% among all participants; 2019/5000) showed a very high titer over the superior limit of quantification (>2080 BAU/mL). Knowledge of immune measures that are statistically associated with protection against disease (‘correlates of protection’) may allow understanding of the response and the possibility of using new vaccines [23]. In a trial conducted by Feng et al. [21], a ChAdOx1 nCoV-19 (AZD1222) vaccine efficacy of 80% against symptomatic infection with a majority of Alpha (B.1.1.7) variant of SARS-CoV-2 was achieved with 506 BAU/mL for anti-spike IgG antibodies. A total of 3606 (72.12%) of the participants showed an equal or superior titer of 506 BAU/mL; thus, a high percentage of the HCWs who participated in the study show a very good protection titers against the Alpha variant according to the data published by Feng et al. [23]. Despite this fact, these data should be reviewed according to the appearance of new variants of concern, such as the Omicron variant [24].

Due to the concurrence of the seroepidemiological study at the beginning of the fifth wave in Spain with the appearance of the Delta variant, we decided to follow up, two months later, with infected HCWs who had been enrolled in the study to address if infection was correlated with total IgG anti-S antibody titers. According to other studies, the coverage in the case of Delta variant infections was more than 90% within 1 month of full vaccination [25], and the mRNA vaccines also showed very good protection against variants other than the Delta variant [25]. This situation changed after the appearance of Omicron. Other reports seem to postulate a significant decline in the effectiveness of full mRNA COVID-19 vaccination, from 74.7% during the pre-Delta period (1 March–9 May 2021) to 53.1% during the period when the Delta variant predominated in the United States [26]. In our setting, the incidence of cases recorded in HCWs in the fifth wave was not very high, representing 1.79% (162 of 9315 HCWs in our area). It can be concluded that, during the fifth month after their implementation, the vaccines seem to have very good effectiveness, as other authors have noted [25,27].

These data support the idea of the prolonged elicitation of antibody response after more than 4 months. Additionally, the infected HCWs in the months after the seroepidemiological study showed median antibody levels lower than those with no infection (*p* < 0.01).

Our data are consistent with other studies that demonstrate an inverse relationship between IgG levels and PCR-positive infection. Kertes et al. [28] found that individuals vaccinated in the first 2 months had a higher probability of being infected than those vaccinated at a later time [28], with serologic values lower than 300 AU/mL (arbitral units per milliliter). The units AU/mL and BAU/mL are not comparable, and in the study of Kertes et al. [28], different antigens were used; hence, these values should not be compared. Despite this fact, this slightly lower threshold according to our results may be explained due to the lack of specific immunoassay correlations or certain particularities of the predominant variants in each study (Alpha vs. Delta). Knowing the concentration of anti-S antibodies is useful for the prediction of the possibility of breakthrough infections, and to provide an evidence-based model of SARS-CoV-2 immune protection [29].

In our population, no differences between ages were observed (*p* > 0.05) compared with the data of Kertes et al. [28], but, in our case, participants > 65 were not included and only eight patients showed a PCR-positive result.

One of the main limitations of our study is that the data were self-reported, which may result in gaps or confusion in the data recorded, including the date of vaccination or other data that were missing in some participants. Additionally, the study population was not randomly selected, as participation in the serological study was optional, so participants may have a greater interest in relation to knowing their level of antibodies. Moreover, the number of participants with positive PCR results was small, so more PCR-positive cases or a longer follow-up period should be added to obtain stronger correlations.

Only in five cases with complete vaccination were antibodies were not detected. In two cases, immunological assays were performed to understand immune memory and response to SARS-CoV-2 vaccines. In both cases, spike protein T reactivity was detected despite not having detected anti-spike IgG antibodies. The absence of antibodies after vaccination can be explained by the alteration found in B lymphocyte compartment and humoral response in both cases. Thus, the SARS-CoV-2 vaccination response based on T-cell reactivity may be an element to consider in the context of booster vaccines in this type of patient, as monitoring of T cell reactivity associated with vaccination may provide important information in terms of protection against infection and disease [15].

The relationship seen in this study with the data obtained in the studied HCWs’ serology and the infection by the Delta variant may not correspond at present with the new epidemiological situation. New studies will be necessary to establish this relationship between infections or reinfections by new variants of concern and anti-S antibody levels.

By the end of 2021, the Omicron variant had emerged across Europe, producing a high rate of infections among fully vaccinated HCWs. This new wave has hit HCWs, in particular, who constitute one of the risk groups identified as being high-risk [30].

Initiatives have been launched for the harmonization of immune response assessment across COVID-19 vaccines [31]. The use of International Standard Units will help in the establishment of a cutoff to determine the need for an additional booster vaccination. Following the implementation of the third and the fourth dose, it now seems plausible that a booster of a fourth dose must be encouraged, especially in vulnerable populations, although not in young health care workers [32].

These data support the idea that knowledge of the concentration of anti-S antibody levels can predict the likelihood of breakthrough infection. Therefore, a booster of the COVID-19 vaccine according to variants of concern that are circulating will be useful in concrete populations and in the case of HCWs.

## Figures and Tables

**Table 1:** Demographic and professional characteristics of the study group.

	Participants *n* (%)
Sex	
Male	959 (19.18)
Female	4019 (80.38)
Not informed	22 (0.44)
Age	
18–34	1309 (26.18)
35–54	2246 (44.92)
≥55	961 (19.22)
Not informed	484 (9.68)
Job Location	
Primary Care *	2556 (51.12)
Tertiary Care **	2444 (48.88)
Health Care position	
Nurse	1681 (33.62)
Physician	914 (18.28)
Health Care Support Services	755 (15.1)
Laboratory Technician	252 (5.04)
Administrative Healthcare	996 (19.92)
Other	395 (7.9)
Not informed	7 (0.14)
Previous COVID-19 Diagnosis	
Yes	828 (16.56)
No	4139 (82.78)
N/A	33 (0.66)
Type of vaccine	
Pfizer	4519 (90.38)
Moderna	285 (5.7)
Others	10 (0.2)
Not informed	186 (3.72)
Vaccination Dosages	
One	166 (3.32)
Complete (2 doses)	4368 (87.36)
Not vaccination	182 (3.64)
Not informed	284 (5.68)

* Primary Care: General practitioner facilities. ** Tertiary Care: Hospital facilities.

**Table 2:** Symptoms classification in SARS-CoV-2 vaccinated participants.

Category	*N*	%
No symptoms	40	47.1
‘Flu-Like’ with fever	26	30.6
‘Flu-Like’ no fever	14	16.5
Gastrointestinal	5	5.9

**Table 3:** Lymphocyte subpopulations and immunoglobulin levels in Anti-SARS-CoV-2 IgG negative participants.

	Participant 1	Participant 2	Reference Values	
			Min.	Max.
Total lymphocytes (cell/uL)	1700	1200	1200	3500
CD19+ lymphocytes (cell/uL)	58	58	100	500
CD19+ lymphocytes (%)	3.4	4.8	6	19
CD3+ lymphocytes (cell/uL)	1027	628	700	2100
CD3+ lymphocytes (%)	60.4	52.3	55	83
CD4+ lymphocytes (cell/uL)	493	416	700	1800
CD4+ lymphocytes (%)	29	34.7	30	50
CD8+ lymphocytes (cell/uL)	332	188	430	1500
CD8+ lymphocytes (%)	19.5	15.7	18	40
IgG immunoglobulin	339	621	700	1400
IgA immunoglobulin	45	150	70	400
IgM immunoglobulin	41	30	40	230
SARS-CoV-2 T cell reactivity	Positive	Positive	Absence	

## Data Availability

Data are available on reasonable request from the corresponding author.
